# Anti-Apoptotic Genes in the Survival of Monocytic Cells During Infection

**DOI:** 10.2174/138920209788920967

**Published:** 2009-08

**Authors:** Aurelia Busca, Mansi Saxena, Marko Kryworuchko, Ashok Kumar

**Affiliations:** 1 Infectious Disease and Vaccine Research Centre, Children’s Hospital of Eastern Ontario, Research Institute, Division of Virology; 2 Department of Pathology and Laboratory Medicine, University of Ottawa, Ottawa, Ontario, Canada; 3 Department of Biochemistry, Microbiology and Immunology, University of Ottawa, Ottawa, Ontario, Canada

**Keywords:** Apoptosis, monocytes/macrophages, HIV, anti-apoptotic genes, tuberculosis.

## Abstract

Macrophages are cells of the immune system that protect organisms against invading pathogens by fulfilling critical roles in innate and adaptive immunity and inflammation. They originate from circulating monocytes and show a high degree of heterogeneity, which reflects the specialization of function given by different anatomical locations. Differentiation of monocytes towards a macrophage phenotype is also accompanied by an increase of resistance against various apoptotic stimuli, a required characteristic that allows macrophages to accomplish their function in a stressful environment.

Apoptosis, a form of programmed cell death, is a tightly regulated process, needed to maintain homeostasis by balancing proliferation with cellular demise. Caspases, a family of cysteine proteases that are highly conserved in multicellular organisms, function as central regulators of apoptosis. FLIP (FLICE-inhibitory protein), anti-apoptotic members of the Bcl2 family and inhibitors of apoptosis (IAP) are the main three groups of anti-apoptotic genes that counteract caspase activation through both the extrinsic and intrinsic apoptotic pathways.

Modulation of the apoptotic machinery during viral and bacterial infections, as well as in various malignancies, is a wellestablished mechanism that promotes the survival of affected cells. The involvement of anti-apoptotic genes in the survival of monocytes/macrophages, either physiological or pathological, will be described in this review. How viral and bacterial infections that target cells of the monocytic lineage affect the expression of anti-apoptotic genes is important in understanding the pathological mechanisms that lead to manifested disease. The latest therapeutic approaches that target anti-apoptotic genes will also be discussed.

## INTRODUCTION

Cells of the monocytic lineage are specialized phagocytes responsible for ingesting and eliminating senescent or infected cells. Macrophages are key players in innate immunity because they respond to infectious microorganisms by producing cytokines that act as mediators of subsequent immune responses. Processing and presentation of antigens also contribute to T cell activation, with direct consequences on the development of both the humoral and cell-mediated immunity. Because of their crucial role in immunity, regulation of monocyte/macrophage life span is important in both physiological and pathological processes.

Apoptosis is a self-destructive cellular process important in tissue development and immune regulation that generally culminates with the sequential activation of caspases, the cysteine proteases responsible for cleavage of specific proteins that ultimately results in cellular demise [1]. Classically, there are two pathways that can result in apoptosis depending on the origin of the apoptotic signal. The extrinsic pathway is initiated by ligand binding to the molecules of the death receptor family whereas the intrinsic pathway gets activated when damage of the mitochondrial membrane causes release of cytocrome c (Cyt-C) in the cytosol and subsequent caspase activation. Both pathways converge with the activation of effector caspases 3 and 7 [2].

**Fig. (1):**
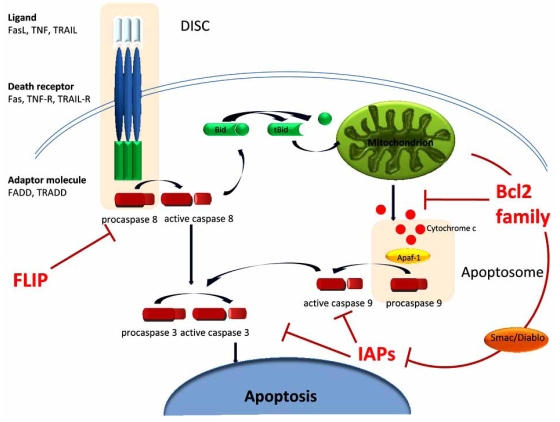
**Overview of apoptosis and the main anti-apoptotic molecules:** Binding of death receptors (Fas, TNF-R, TRAIL-R) to their ligands (Fas-L, TNF, TRAIL) initiates the extrinsic apoptotic pathway. Association of adaptor molecules (FADD - Fas-associated death domain protein, TRADD–TNF receptor associated death domain protein) induces the formation of DISC (death inducing signaling complex), which activates caspase 8 and subsequently caspase 3. FLIP is the main anti-apoptotic molecule of this pathway, as it prevents caspase 8 activation. Release of cytochrome c from the mitochondria in response to cellular stress initiates the intrinsic apoptotic pathway. Cytochrome c associates with Apaf-1 and procaspase 9 to form the apoptosome, a multimeric protein complex that induces cleavage of inactive caspase 9 to its active form. Both pathways converge with activation of caspase 3, the main effector caspase. The two pathways are also connected by the ability of active caspase 8 to activate Bid (BH3-interacting domain death agonist) – tBid (truncated Bid) is a proapoptotic molecule that can induce release of cytochrome c from the mitochondria and thus initiate the intrinsic pathway. The two main antiapoptotic families are Bcl2 and IAPs. Anti-apoptotic Bcl2 members maintain mitochondrial integrity, while IAP can inactivate caspases. IAP activity is antagonized by Smac (also known as Diablo), a proapoptotic molecule released from the mitochondria [1, 9, 12].

There are three classes of anti-apoptotic proteins that act at different stages throughout caspase activation to counteract death-inducing signals and prevent over-activation of the apoptotic machinery (Fig. **1**).

1. The FADD-like IL-1β converting enzyme (FLICE) inhibitory protein (FLIP) is the main anti-apoptotic mechanism in the extrinsic pathway. It prevents activation of caspase 8 (also known as FLICE) following ligation of death receptors like Fas and the TNF-related apoptosis inducing ligand (TRAIL) receptors and subsequent cleavage of effector caspases (e.g. caspase 3) [3]. Due to structural homology with caspase 8, FLIP is able to bind and form FLIP-FLICE heterodimers that prevent subsequent activation of FLICE [4].
2. The anti-apoptotic proteins of the Bcl2 family (e.g. Bcl-xL and Bcl2) maintain the integrity of the mitochondrial membrane and prevent activation of caspases due to Cyt-C release [5, 6]. The Bcl2 family also contains pro-apoptotic members such as Bax and Bak that promote apoptosis by binding and inactivating their anti-apoptotic counterparts [7]. The balance between the two groups ultimately dictates cell fate.
3. The third group comprises the family of inhibitors of apoptosis (IAPs) proteins. These proteins are the major regulators of cell survival because they act on caspases activated either through the extrinsic or the intrinsic pathway. Initially discovered in baculoviruses as an *iap* gene [8], there are now eight mammalian IAPs: cellular IAP1 (c-IAP1), c-IAP2, X-chromosome-linked IAP (XIAP), neuronal apoptosis inhibitory protein (NAIP), survivin, livin, IAP-like protein 2 (ILP2) and baculovirus inhibitor of apoptosis repeat containing ubiquitin-conjugating enzyme (BRUCE) [9]. IAPs share variable numbers of baculoviral IAP repeat (BIR) motifs, structural domains that are important for binding and inactivation of both initiator and effector caspases [10, 11].

As monocytes differentiate into macrophages, they also increase their resistance to spontaneous and induced apoptosis, a beneficial mechanism during immune responses against pathogens. Enhanced survival of macrophages is even more important in various pathological conditions in which cells of the monocytic lineage are key players such as infections with intracellular viral and bacterial pathogens, inflammatory conditions and monocytic malignancies, where the enhanced survival of this cell type is no longer beneficial and becomes a main factor in pathogenesis. Apoptosis is a very important weapon of host immunity against intracellular pathogens like Human Immunodeficiency Virus (HIV) and *Mycobacterium tuberculosis* (M.tb). Apoptosis of infected cells serves several following purposes: 1) killing or reducing the viability of intracellular pathogens, 2) preventing dissemination of the microbes, 3) providing other antigen presenting cells (APCs) with microbial antigens in apoptotic bodies and 4) preventing persistence and formation of reservoirs [12]. Various arguments and evidence suggest that intracellular pathogens may evade apoptosis of infected monocytic cells by up regulating various host anti-apoptotic genes that dysregulate both extrinsic and intrinsic apoptotic pathways in these cells. In this review we will discuss the role of these anti-apoptotic proteins in the increased survival of macrophages in both physiological and pathological conditions, with an emphasis on HIV and M.tb. infections, intracellular pathogens that target cells of phagocytic system.

## ROLE OF ANTI-APOPTOTIC GENES IN HEMATOPOIESIS

Members of the Bcl2 family have been shown to be differentially implicated in hematopoiesis of the myeloid lineage. Granulocytes and monocytes/macrophages are two distinct lineages that originate from a common myeloid precursor. *In vitro* studies with CD34+ progenitor cells [13] and the promonocytic cell line HL60 [14] induced to differentiate by chemical agents revealed an increased expression of Bcl-xL in cells committed to the monocyte/macrophage lineage, but not when cells were induced to differentiate to granulocytes. Bcl-xL upregulation throughout the monocytic lineage is accompanied by down regulation of anti-apoptotic Bcl2 protein [15-17] suggesting divergent roles among anti-apoptotic members of this family in determining the enhanced lifespan of monocytes over granulocytes.

Differential involvement of Bcl2 and Bcl-xL in hematopoiesis is also illustrated in mouse model studies. Bcl-xL knockout mice die during embryogenesis with massive apoptosis of cells of the hematopoietic and central nervous system [18]. In contrast, Bcl2 knockout mice are born with organ malformations but they survive without major disruptions in hematopoiesis [19]. These studies suggest that while Bcl2 is necessary for normal morphogenesis, Bcl-xL is vital for hematopoiesis. Interestingly, when macrophages are obtained from immature bone marrow precursors cultured in the presence of M-CSF, Bcl2 expression shows a different pattern, being upregulated in both human [20] and mouse models [20, 21]. Although the expression of Bcl-xL was not examined in these studies, one possible explanation for these divergent results would be that immature bone marrow precursors are highly susceptible to apoptosis and require M-CSF for survival, which may trigger a different pattern of anti-apoptotic gene(s) expression in order to overcome higher susceptibility to apoptosis.

## ANTI-APOPTOTIC GENES INVOLVED IN MONOCYTE TO MACROPHAGE DIFFERENTIATION

Monocytes migrate from the blood stream to inflammatory sites where they differentiate into macrophages [22]. During differentiation, monocytes lose their ability to proliferate but increase their phagocytic and enzymatic capacity to become terminally differentiated cells [23]. This process affects not only their function, but also their susceptibility to apoptosis. Monocytes have a short lifespan when cultured *in vitro*. Even in the presence of growth factors, these cells survive only for a few days [24]. However, differentiated macrophages become resistant to both spontaneous and induced apoptosis [25], a characteristic necessary for them to perform their functions in a stressful microenvironment while fighting invading pathogens.

Although an enhanced resistance to apoptosis of monocyte-derived macrophages (MDMs) is known for many years [26], the precise molecular mechanisms are still not fully elucidated. Monocytic cells, unless activated by various stimuli such as inflammatory cytokines or growth factors, undergo spontaneous apoptosis when cultured *in vitro* [26-29]. Activated monocytes are more resistant to various apoptotic stimuli such as death receptor ligands (Fas-L, TNF) [30, 31], reactive oxygen species [32], DNA damage [33], and proteasome inhibitors [34, 35]. While the extrinsic apoptotic pathway was responsible for spontaneous apoptosis of monocytes through activation of the Fas-Fas ligand pathway, this interaction seemed not to be operating in macrophages, suggesting that protection is the result of events downstream of the death receptors [24]. Interestingly, our recent results have suggested that spontaneous cell death occurs in primary monocytes but depends on the autophagy (“cellular self eating”) pathway rather than on classical apoptosis [36]. Spontaneous cell death could not be blocked by neutralizing TRAIL and Fas death receptors antibodies, or by a general caspase inhibitor. However, this spontaneous cell death was diminished significantly with the autophagy inhibitors 3-methyladenine and chloroquine. Moreover, LC3-II expression, an autophagy marker, was upregulated spontaneously in cultured monocytes. At the molecular level, we observed that blocking the Signal Transducer and Activator of Transcription (STAT) or phosphatidylinositol-3-kinase (PI3K)/ AKT signalling pathways inhibited LC3-II expression and cell death in response to IFN-γ [36].

To unravel the mechanisms of protection in activated monocytes, the apoptotic pathway affected by monocyte activation has been investigated. Perera *et al*. showed that LPS activation induced the expression of anti-apoptotic *Bfl-1* gene of the Bcl2 family and decreased caspase 8 expression suggesting their role in enhanced survival of activated monocytes in response to various apoptotic stimuli [33]. Subsequently, Perlman *et al*. demonstrated that the resistance of MDMs to spontaneous and Fas-FasL mediated apoptosis was attributed to the upregulation of FLIP anti-apoptotic protein throughout differentiation [25]. An enhanced transcription of the *FLIP* gene was detected on day 3 of differentiation and its role was confirmed using antisense oligonucleotides that were able to abrogate the resistance to Fas-L apoptosis by inhibiting the activation of caspase-8. Furthermore, differentiated MDMs require constitutive activation of NFkB [37, 38] and PI3K/Akt pathways [39] in order to sustain their viability. These two pathways independently induce the sustained expression of two different genes of the Bcl2 family, *A1* for NFkB and *Mcl-1* for phosphatidylinositol-3-kinase (PI3K)/Akt pathway, both of which are responsible for maintaining mitochondrial integrity.

In addition to *FLIP* and genes of the *Bcl2* family, monocyte to macrophage differentiation has also been shown to affect the third group of anti-apoptotic genes, *IAPs.* XIAP has been shown to be up-regulated in many experimental models of monocyte differentiation: human [20] or mouse [21] bone marrow-derived macrophages, human macrophages differentiated from promonocytic cell lines in the presence of PMA [16, 40] or from THP1 cells in the presence of bryostatin-1 [35], its upregulation being responsible for the enhanced resistant phenotype of macrophages.

Recently, a new mechanism of anti-apoptotic gene involvement in monocyte differentiation has been proposed. Plenchette *et al*. demonstrated that c-IAP1 translocates from the nucleus to the Golgi apparatus in both mouse and human monocytes induced to differentiate into macrophages. There was no nucleo-cytoplasmic redistribution of cIAP1 in cells whose differentiation was prevented, such as in Bcl2 overexpressing cells and monocytes from patients with chronic myelomonocytic leukemia [41]. The functional nuclear export signal was restricted to its caspase recruitment domain (CARD), a motif that is classically involved in protein interactions and caspase aggregation [42]. Although caspase activation was not evaluated during c-IAP1 translocation, the same group also showed that monocytic cells undergoing macrophage differentiation require a basal level of caspase-3 and -9 activation that does not result in apoptosis, but is nevertheless essential for optimal differentiation [43-45], thus extending our knowledge about the key role of caspases in monocyte differentiation (for a review see [46]). The contribution of anti-apoptotic genes in the regulation of caspase activation during monocyte differentiation is yet to be determined. However, these observations suggest a relationship between various levels of anti-apoptotic genes that are known to prevent caspase activity and the activation status of caspases in monocytes undergoing differentiation. Overexpression of the Bcl2 gene has been shown to inhibit differentiation of U937 monocytic cell line in the presence of phorbol ester [43] or bleomycin [47], so it is tempting to speculate that the mechanism of this inhibition would be prevention of caspase activation.

These observations suggest the existence of multiple seemingly redundant mechanisms directed at preventing the release of Cyt-C from the mitochondria, a crucial event of apoptosis that is controlled by Bcl2 proteins. However, the mechanism responsible for the differential involvement of some members of the Bcl2 family over others in regulating macrophage survival remains unknown. Modulation of anti-apoptotic genes that act at various stages of the apoptotic pathway seems to be the main mechanism by which differentiation confers macrophages enhanced surviving abilities. This complex process does not involve just one key player or anti-apoptotic molecule, but multiple mechanisms that vary with the nature of the stress signal, a situation that is to be expected for a cell type that is often the first line of defense in immune responses.

## SURVIVAL OF MONOCYTE/MACROPHAGES IN HIV INFECTION

### Viral Reservoirs: Lymphocytic Versus Monocytic Cells

Along with CD4+ T cells, cells of the monocytic lineage represent a major viral reservoir during HIV infection [48, 49]. However, there are major differences between virus biology within these two cellular systems: viral replication in T cells is highly cytopathic and as the virus replicates and the disease state advances, the number of CD4+ T cells is ultimately reduced. The formation of viral reservoirs in T cells is correlated with the antigen-induced T cell activation and proliferation. The infected lymphocytes that survive the viral cytolytic effect return to a resting state which corresponds to memory T cell establishment [50] which is incapable of supporting active viral replication. Since these cells contain integrated virus, viral replication can be reactivated by stimulation with the cognate antigen [51-53]. It seems that the virus adapts itself perfectly for long-term survival by hijacking the normal mechanism of long-lived memory cell establishment. Hence, there is no clear evidence to suggest that latency in CD4+ T cells has evolved as a viral mechanism to promote persistence in this cell type. On the other hand, macrophages get infected early during infection and survive active viral replication [54, 55], serving as a continuous source of viral progeny especially during the late phases of infection, when CD4+ T cells are being lost [56]. Investigations on viral reservoir formation have received a lot of attention, most of it being focused on memory T cells and the regulation of viral transcription [57]. However, it is clear that different mechanisms operate in T cells and monocytic cells, susceptibility to the virus cytopathic effect being one of them.

As mentioned, monocyte to macrophage differentiation induces an increased resistance to a multitude of apoptotic stimuli. However, comparative studies of monocyte and macrophage susceptibility to apoptosis in the context of HIV infection have been hampered by the monocytes’ lack of susceptibility to *in vitro* HIV infection [58, 59]. Monocyte differentiation increases their permissiveness to viral replication, with macrophages making up a primary source of HIV reservoirs [60]. Persistently-infected monocytes have also been isolated from the peripheral blood of HIV-infected patients in particular those under antiretroviral treatment [61-63] suggesting that lack of susceptibility to infection of monocytes *in vivo* is not absolute.

## ROLE OF BCL2 IN RESISTANCE TO APOPTOSIS IN HIV-INFECTION

### Evidence from Cell Lines

It is now clear that HIV affects the apoptotic pathways differently in monocytic and lymphocytic cell lines chronically infected with HIV. Chronically infected monocytic U1 cells were found to be more resistant to apoptosis induced by γ-irradiation or TNFα plus cycloheximide compared to the chronically infected lymphocytic cell line ACH-2. At the same time, the Fas/FasL death receptor pathway was less functional in both cell lines compared to their uninfected counterparts [64]. Although the mechanisms responsible for the discrepancies between the two cell types were not investigated, these results confirmed earlier observations on the decreased sensitivity to death receptor-induced apoptosis of HIV-infected monocytic cells [65, 66]. Even though the mechanism underlying increased apoptosis resistance of persistently infected monocytic cells is not clear, it is reasonable to hypothesize that differential expression of anti-apoptotic molecules may contribute to this resistance. Recently, Fernandez-Larrosa *et al. *[67] showed that the increased resistance to staurosporine- and H_2_O_2_-induced apoptosis in chronically HIV-infected monocytic cell lines was modulated through the mitochondrial pathway, with an increased Bcl2/Bax ratio in the infected cells favoring an anti-apoptotic phenotype. More importantly, this resistance to apoptosis was independent of the magnitude of viral replication, which suggests that controlling the apoptotic pathway is a major factor that influences viral persistence beyond continuous replication.

### Evidence from Primary Cells

The involvement of Bcl2 family in increased survival of macrophages during HIV infection was also confirmed in studies that used *in vitro* infection of MDMs [68, 69]. In this model, HIV infection increased the expression of anti-apoptotic Bcl2 and Bcl-xL and decreased proapoptotic Bax and Bad proteins [69]. We have recently shown that spontaneous cell death and IFN-γ-induced monocyte cell death was elevated in HIV+ patients compared to HIV- controls [70]. Interestingly, the anti-inflammatory cytokine IL-10 was able to reduce spontaneous and IFN-γ-induced monocyte cell death in both normal and HIV-infected patients (unpublished observations). However, a recent study demonstrated that monocytes from HIV+ patients under different conditions (CdCl_2_ and Fas) are more resistant to cell death compared to uninfected controls [71], even in the absence of productive infection. Even though the expression of Bcl2 and IAP families was not evaluated, the enhanced resistance of monocytes isolated from HIV+ donors was linked with the virus ability to bind and activate CCR5, the main coreceptor for macrophage tropic viruses [72]. These results indicate that the effects of viral infection may extend beyond infected cells, and highlight the complicated role of apoptosis in HIV pathogenesis.

Several HIV proteins such as Tat, Nef and Vpr can modulate the survival of monocytic cells depending on their stage of differentiation and through the expression of a variety of cytokines such as MIP-1 α, β and other proinflammatory TNF-α or IL-6 cytokines.

#### HIV Tat

The macrophages not only resist the cytopathic effect of HIV [73] but also contribute to an increased cell death of uninfected bystander CD4 T cells by upregulating the expression of FasL and interacting with Fas-expressing lymphocytes [74, 75] or by increasing TRAIL secretion [76]. The ability to contribute to lymphocyte apoptosis has also been demonstrated for monocytes. Consistent with bystander cell apoptosis, treatment of monocytes with HIV-Tat resulted in an increased secretion of TRAIL in the culture media which is more cytotoxic for uninfected than the CD4+ HIV-infected T cells [77]. Interestingly enough, treatment of monocytes with HIV Tat was also shown to induce increased expression of Bcl2 which was able to protect monocytes against TRAIL-induced apoptosis [78]. The ability of Bcl2, a protein associated with maintaining mitochondrial membrane integrity and anti-apoptotic activity through the intrinsic pathway, to protect against receptor-mediated cell death (extrinsic pathway) shows the complexity of cell survival regulation in this cell type. This may be the reason why the virus has evolved pathways to target this protein family to its advantage.

#### HIV Nef

The Bcl2 family has also been implicated in the enhanced survival of macrophages in response to HIV-Nef [79, 80], an accessory protein known to protect lymphocytes from HIV cytopathic effects by inducing phosphorylation and inactivation of pro-apoptotic Bad [81]. Although other anti-apoptotic genes were not evaluated and a direct cause and effect relationship was not established, Nef expression was correlated with increased Bad phosphorylation and cell survival in a VSV-G HIV pseudovirus infection model of MDMs [79]. Similarly, Nef expression increased the survival of a TF-1 macrophage precursor cell line after cytokine removal by enhancing Bcl-xL gene expression [80]. Recently, the ability of HIV-infected macrophages to resist TRAIL-mediated apoptosis was found to be associated with induction of the Bcl2 family of genes [82]. Although HIV infection up-regulated a number of anti-apoptotic genes such as cIAP1, cIAP2, XIAP, Mcl-1 and Bfl-1, only Mcl-1 and Bfl-1 protected HIV-infected macrophages against TRAIL-induced apoptosis. Moreover, this resistance was found to be dependant on secretion of M-CSF, a cytokine known to stimulate HIV replication [83]. In addition, this resistance to apoptosis was not detected in HIV strains deficient for the envelope gene [82].

Overall, it seems that Tat and Nef have overlapping activities in terms of promoting survival of infected cells especially by targeting Bcl2 family of proteins (results summarized in Table **1**). Although results depend on different experimental settings and the models used to deliver viral proteins, it is reasonable to conclude that modulation of the apoptotic pathway contributes to maintenance of viral reservoir status. The implications may also be extended to bystander uninfected cells, given the ability of infected cells to secrete viral proteins such as Tat in the extracellular medium [84].

#### HIV Vpr

Despite the presence of a low number of infected monocytes under *in vivo *conditions, monocytes can be affected by viral proteins that are secreted in the patient’s serum, suggesting that experimental approaches using viral peptides may yield more relevant results. In order to study susceptibility to apoptosis in the context of HIV in a manner that would circumvent the lack of* in vitro *productive infection, we have used Vpr (viral protein R) as an experimental model for monocyte apoptosis. Vpr has been shown to cause apoptosis in several cell types, including lymphocytes [85, 86], monocytes [87] and neurons [88-90]. By using a synthetic peptide encoding the C-terminal 52-96 amino acid sequence of Vpr, which contains the apoptosis inducing domain [91], we investigated the effect of Vpr in primary human monocytes and in THP1 promonocytic cell line. Our results show that Vpr52-96 induced apoptosis in both cell types was caspase dependant and involved the activation of c-Jun-N-terminal kinase (JNK) mitogen-activated protein-kinase (MAPK) [87]. Vpr also down regulated the expression level of Bcl2 and cIAP1 anti-apoptotic proteins downstream of JNK activation. After inhibition of the JNK pathway with pharmacological inhibitors and siRNA, both the apoptotic effect and the decreased expression of Bcl2 and cIAP1 were reversed, which linked the effect on monocyte survival to Vpr’s ability to modulate the expression level of these genes.

Our preliminary results suggest that the Vpr-induced apoptotic effect could be inhibited by pretreatment of monocytes with certain Toll-like receptor (TLR) agonists such as LPS (TLR-4), PolyI:C (TLR-3), CpG (TLR-9) or the proinflammatory cytokine TNF-α. Furthermore, LPS and TNF-α protected monocytic cells against Vpr-induced apoptosis by upregulating the cIAP2 gene, suggesting that different mechanisms are operative in regulating sensitivity/resistance to Vpr-induced apoptosis (unpublished observations). Moreover, we have also demonstrated the involvement of cIAP2 in LPS-induced protection of monocytes from staurosporine-induced apoptosis [92] indicating that this anti-apoptotic gene may play a key role in monocytic cell survival against multiple apoptotic stimuli. However, monocytic cells obtained after differentiation as MDMs were no longer responsive to the Vpr-induced apoptotic effect (unpublished observation) suggesting that differentiated macrophages possess an enhanced survival phenotype that characterizes these cells during HIV infection. Our results also suggest that resistance to apoptosis is linked to Vpr’s inability to down regulate anti-apoptotic molecules in macrophages. If this is the case, it is tempting to hypothesize that targeting anti-apoptotic genes would provide a valuable therapeutic tool in eliminating this viral reservoir.

## TUBERCULOSIS INFECTION AND ANTI-APOPTOTIC GENES: THE HOST’S PERSPECTIVE

It is well established that M.tb establishes persistence in infected macrophages by exploiting their phagocytic machinery and by preventing acidification of phagolysosomes [93]. Reports of host autophagic machinery being possibly used by M.tb for the same purpose has been also been recently elucidated [94]. Another major mechanism evolved by M.tb to establish persistence is prevention of apoptosis in infected macrophages and monocytes [93-96]. There is ample evidence to suggest that M.tb, like HIV, evades apoptosis of infected monocytic cells by upregulating several host’s anti-apoptotic genes responsible for dysregulating both extrinsic and intrinsic apoptotic pathways.

## INDUCTION OF PRO- AND ANTI-APOPTOTIC GENES BY VIRULENT AND AVIRULENT M.TB STRAINS

Literature on whether virulent M.tb induces or prevents apoptosis in infected cells has been somewhat controversial [97]. Studies using different mouse strains, cells and systems have reported variable results. Some studies claim that M.tb infection induces apoptosis in infected macrophages [98]. However, there are equally convincing reports of M.tb inducing protection against apoptosis in infected cells [93, 96, 99]. Even though differences in mode of infection, experimental techniques and cells used may account for some of these differences, one of the important reasons behind these discordant results is the differential regulation of apoptotic genes by virulent, avirulent and attenuated strains of M.tb.

Katarzyna-Sablinska* et.al. *(1998) demostrated that virulent and avirulent M.tb strains induce different types and degrees of cell death in infected macrophages [95]. Both, avirulent H37Ra and virulent H37Rv strains induce apoptosis in infected human alveolar macrophages (AMs) [100]. However H37Ra infection in U937 cells induced higher levels of apoptosis compared to the H37Rv infected cells that showed signs of necrosis [93, 96]. Similarly, extremely virulent Beijing strain of M.tb was shown to induce even less apoptosis than H37Rv strain and strongly induced necrosis in THP1-derived macrophages and in murine macrophages [101, 102]. Why virulent M.tb strains would drive host cells towards necrosis can be explained by the fact that apoptosis of host cells severely reduces bacterial viability whereas necrosis has no effect on bacterial viability as confirmed by colony forming units (CFUs) formed by monocytic cells infected by H37Ra and H37Rv strains [103].

The stage of cell differentiation also determines whether M.tb infection will induce apoptosis or protection. Monocyte derived macrophages from healthy tuberculin positive donors are more prone to undergo M.tb-induced apoptosis as compared to monocytes from the same donors [104]. Moreover, U937 cells treated with purified protein derivatives (PPDs) underwent necrosis whereas U937-derived macrophages died of apoptosis upon PPD treatment [99]. Another factor that may influence induction of apoptosis or necrosis in monocytic cells infected by M.tb is the bacterial dose or multiplicity of infection (m.o.i). Murine macrophages infected with H37Rv at high m.o.i of >25 protected the infected cells from apoptosis and induced necrosis whereas infection with low m.o.i, of <10 of the same strain induced TNF-α-induced apoptosis [105].

Both virulent and avirulent M.tb strains induce apoptosis in monocytic cells when compared to uninfected controls. In general, the avirulent strains induce much higher levels of apoptosis than the virulent strains (90). However, analysis of microarray of apoptosis-related genes in monocytic cells infected with virulent and avirulent strains have revealed variable results. Some groups show upregulation of anti-apoptotic genes whereas others demonstrate induction of pro-apoptotic genes. For example, apoptosis pathway(s) specific cDNA microarray analysis of U937 cells infected with virulent M.tb strain, H37Rv, revealed that anti-apoptotic genes, *Bcl-2* and *Rb* (retinoblastoma 1), and caspase-1 were down regulated, whereas pro-apoptotic *p53 *oncogene*, Bad *and* Bax *genes were significantly upregulated by 48 hrs in infected cells as compared to the uninfected cells [96]. Spira *et al*. also conducted a gene microarray to profile apoptosis-related genes induced in alveolar macrophages (AMs) infected with avirulent H37Ra and virulent H37Rv strains. They reported that despite a unique genetic profile for each donor, certain discrete pro-apoptotic genes, *Zk1, FIP3* and *EF-1alpha,* were significantly and consistently down regulated in AMs infected with H37Rv as compared to the H37Ra-infected cells. Moreover, the anti apoptotic *Bcl-w* gene was found to be upregulated in H37Rv infected AMs as compared to the uninfected cells [106]. In contrast, another study showed upregulation of pro-apoptotic *casp10* and *rps19* genes, and anti-apoptotic *Bcl2l1* gene in U937 cells infected with H37Rv strain [107]. Several members of the anti-apoptotic NFκB gene family, *NFκB1, NFκB2, IκBA, REL, RELA, RELB* were upregulated by virulent H37Rv strain in primary human monocytes [108]. Taken together, these reports suggest that even though virulent and avirulent M.tb strains induce both pro and anti-apoptotic genes in infected macrophages, the virulent strains are able to inhibit or alter the apoptotic functions of induced pro-apoptotic genes.

NFκB is a transcription factor that regulates several anti-apoptotic genes including certain IAPs and members of Bcl2 family [20]. NFκB has been shown to translocate to the nucleus following M.tb infection in macrophages [109]. THP1-IκBαMdn cells, the cells lacking NFκB, infected with virulent M.tb undergo down regulation of anti-apoptotic gene, *A1* [110]. *A1* is an NFκB-dependant anti- apoptotic gene, belonging to the Bcl2 family of proteins, that was first characterized in mice and its human homologue was identified in HUVEC cells by Karsan *et al.* [111]. The *A1*α isoform was found to mediate protection against nitrous oxide-induced apoptosis in BCG infected murine macrophages and in J774, a murine macrophage cell line. The upregulation of isoform* A1a* was shown to be independent of protein synthesis, p38 MAP Kinase and PI3K signaling pathways. Moreover, macrophages from A1α knockout mice died from nitrous oxide-induced apoptosis [112]. These results were confirmed in human model system wherein H37Rv and H37Ra induced differential expression of A1 in THP-1 cells and in MDMs. Abrogation of A1 by siRNAs significantly reduced H37Rv-induced protection which resulted in reduced bacterial viability in THP1 cells [113]. Another member of the Bcl2 family, Bad, was shown to be involved in enhanced survival of human monocytic cells infected with M.tb. Bad needs to be dephosphorylated in order to become active following which it translocates to the mitochondria prior to inducing apoptosis [114]. Mycobacterial mannosylated Lam from virulent strains of M.tb was shown to phosphorylate Bad in a PI3K/Akt dependant manner in THP1 cells thereby avoiding yet another host apoptotic mechanism [115]. Similarly, NFκB-regulated Myeloid cell factor-1 (*Mcl-1*), an anti apoptotic member of the Bcl2 family of proteins has been reported to promote monocytic cell survival against M.tb infection [116]. Mcl1 serves to preserve integrity of mitochondrial membrane, prevent release of mitochondrial apoptotic factors and inhibit induction of effector caspases [2]. *Mcl-1* expression is up regulated in THP-1 cells and MDMs macrophages infected with live, virulent M.tb strain, H37Rv [116, 117]. Moreover blocking *Mcl-1* expression using siRNA significantly increases apoptosis in H37Rv infected cells [116]. A study conducted on Korean males also proposes a strong correlation between Mcl-1 polymorphism and disease susceptibility [117].

## ROLE OF TNF-Α AND TOLL-LIKE RECEPTORS (TLRS) IN M.TB-INDUCED ANTI-APOPTOTIC GENES AND APOPTOSIS

The anti-apoptotic genes induced by virulent M.tb appear to reduce apoptosis by interfering with the TNF-α-induced extrinsic apoptotic pathway. Neutralizing TNF-α in H37Ra-infected cells converts their gene profile to that of H37Rv-infected cells [95, 96, 105, 106]. Besides, virulent M.tb strains have been shown to induce shedding of soluble TNFR2, consequently inhibiting TNF-α-induced apoptosis [95]. Virulent M.tb reduces expression of Fas on the cell surface of infected human macrophages presenting yet another mechanism of evading host cell apoptosis by abolishing Fas/FasL mediated apoptosis [103].

M.tb also exploits TLRs to induce anti-apoptotic genes that enhance cell survival and promote bacterial persistence [109]. Exploiting TLRs is not a mechanism unique to M.tb. As mentioned earlier, we have shown that TLR3, TLR4 and TLR9, when stimulated by their ligands PolyI:C, LPS and CpG DNA, respectively, protected monocytic cells from HIV-Vpr induced apoptosis by induction of NFκB and anti-apoptotic cIAP genes (unpublished data). Stimulation of TLR2, found in abundance at sites of M.tb infection, by components of M.tb cell wall, has been shown to protect human macrophages against apoptosis. THP1-derived macrophages when stimulated with 19kDa mycobacterial lipoprotein or mannosylated LAM were shown to induce resistance to apoptosis *via *activation of NFκB and subsequent induction of anti apoptotic *cFLIP* which inhibits death receptor-mediated apoptosis [25, 109].

## ANTI-APOPTOTIC GENES AS POTENTIAL DRUG TARGETS

Understanding how anti-apoptotic proteins contribute to the enhanced survival of infected macrophages has a direct impact on therapeutic strategies. For example, IAP and Bcl2 antagonists are currently being developed as cell death inducing therapies in cancer cells, where expression of anti-apoptotic proteins is a major mechanism of pathogenicity and chemotherapy resistance [118, 119]. Smac (second mitochondria-derived activator of caspase), a IAP binding protein released from the mitochondria in response to apoptotic stimuli, promotes cell death by binding and inactivating IAPs [120]. Smac mimetics are chemical compounds that display the same activity in the absence of mitochondrial release of apoptotic factors [118], thereby releasing caspases from IAP blockage and causing apoptosis. Smac mimetics have the ability to promote ubiquitination and subsequent degradation of various IAPs [121], which also affects the signalling pathway of TNF-α, because of IAPs association with the TNF receptor complex [122]. Recent evidence implicates this pleiotropic cytokine as being responsible for the apoptotic effect of Smac mimetics, as only cell lines that secrete TNF-α are sensitive to their effect [121, 123]. TNF-α can generate two different signals through distinctive receptors: one that triggers apoptosis and another that inhibits apoptosis [124]. There is evidence to suggest that it promotes cell survival during HIV infection [69]. Therefore, it is reasonable to hypothesize that HIV infected cells may be sensitized to Smac mimetic apoptosis because they already secrete TNF-α. Other apoptosis modulators of the Bcl2 family are Bcl2 inhibitors [125] and the recently discovered Bcl-xL inhibitors [126] that also prevent protein-protein interactions by binding to the surface pocket that is required for their anti-apoptotic function [125, 126], causing mitochondrial permeability and caspase activation in treated cells.

Both Smac mimetics [121, 123, 127-129] and Bcl-2 family inhibitors [130-132] are intensely studied as potential antineoplasic drugs, but the potential therapeutic benefits of these agents in the context of HIV or M.tb. infections are currently unknown; yet, preliminary evidence presented here indicates that upregulation of anti-apoptotic molecules documented in HIV and M. tb. infections may also sensitize infected cells to the respective antagonists, opening new possibilities in terms of targeting persistently infected cells. Targeting macrophage reservoir during HIV infection may have therapeutic benefits on T cells as well, since infected macrophages have been shown to be responsible for by-stander effect apoptosis on neighbouring cells [74-76].

## CONCLUSIONS

Modulation of the apoptotic pathway is a powerful mechanism for increased survival of monocytic cells, a cell type that is preferentially targeted during HIV and M. tb infections. Even though the extent of its in vivo significance needs to be studied further, in vitro studies have shown that the enhanced resistance of macrophages against various apoptotic stimuli is a complex process that involves modulation of anti-apoptotic genes. Even if the pattern of gene expression may vary with the experimental model, the chemical inducers employed for activation of monocytes or the nature of the apoptotic signal used to challenge differentiated macrophages, the overall tendency is for an increased expression of anti-apoptotic genes as a common mechanism of increased macrophage viability rather than the decreased expression of anti-apoptotic molecules. The genes most frequently involved in macrophage resistance were Bcl2 and Bcl-xL of the Bcl2 family and XIAP of IAP family.

In addition to the increased resistance phenotype displayed by macrophages compared to monocytes, studies also suggests that HIV and M. tb further promote the survival of this cell type by interfering with the expression of anti-apoptotic molecules. Preliminary data involve members of the Bcl2 family as being the main targets in HIV infection, while the data on IAPs involvement in still lacking. Depending upon m.o.i, strain and stage of host cell differentiation, M.tb induces several anti apoptotic genes, including cFLIP and several members of Bcl2 family, like Mcl-1 and A1. While enhanced expression of anti-apoptotic proteins may be deleterious for infected cells, they may also provide a target for apoptosis inducing agents, like Smac mimetics and Bcl2 inhibitors that act specifically on these proteins. Although further studies are needed to evaluate their efficiency in infections, these compounds constitute a novel direction of approaching persistently infected cells, a direction that deserves further consideration.

## Figures and Tables

**Table 1:** The Effect of HIV Infection and Viral Proteins on the Expression Levels of Pro/Anti-Apoptotic Genes in Monocytic Cells. The Impact on Cell Survival or Death, the Molecular Mechanisms Implicated (when Investigated) and the References are also Indicated

Experimental Setting	Cell Type	Molecules Involved	Effect		Mechanism	References
*in vitro* HIV infection, HIV Tat protein	human monocyte derived macrophages	Bcl2	upregulation			[68]
*in vitro* HIV infection	human monocyte derived macrophages	Bcl2 Bcl-XL	upregulation	increased survival of infected cells	increased TNFα secretion in culture media	[69]
		Bax Bad	downregulation			
expression of HIV-Nef by retroviral transduction	TF1 macrophage precursor cell line	Bcl-XL	upregulation	increased survival of Nef expressing cells to cytokine removal	Erk/MAPK signaling	[80]
VSV pseudotyped-HIV infection, *Δnef *clones	human monocyte derived macrophages	Bad	increased phosphorilation	protection of infected cells against spontaneous apoptosis	TNF independent	[79]
Vpr synthetic peptide	primary monocytes, THP1 cells	Bcl2 cIAP1	downregulation	apoptosis	JNK signaling pathway	[87]
TNFα, TLR3-, TLR4-, TLR9- agonists	primary monocytes, THP1 cells	cIAP2	upregulation	protection against Vpr-induced apoptosis		unpublished
*in vitro* HIV infection	human monocyte derived macrophages	Bfl-1 Mcl-1	upregulation	protection against TRAIL-induced apoptosis	- increased MCSF production - downregulation of TRAIL-R1	[82]
HIV Tat protein	primary monocytes	Bcl2	upregulation	protection against TRAIL-induced apoptosis		[78]
